# Different associations of general and abdominal obesity with upper and lower extremity artery disease among a community population in China

**DOI:** 10.1186/s12986-023-00736-1

**Published:** 2023-03-09

**Authors:** Yong Wang, Xiaoyan Guo, Yi Zhang, Ruiyan Zhang, Jue Li

**Affiliations:** 1 Department of Cardiovascular Medicine, Ruijin Hospital, Shanghai Jiao Tong University School of Medicine, 197 Rui Jin 2nd Road, Shanghai, 200025 China; 2 Department of Gastroenterology, Gongli Hospital of Pudong New District of Shanghai, Shanghai, 200135 China; 3 Department of Cardiology, Shanghai Tenth People’s Hospital, Tongji University School of Medicine, Shanghai, 200072 China; 4 Department of Epidemiology, Tongji University School of Medicine, Shanghai, 200092 China

**Keywords:** Atherosclerotic cardiovascular disease, Peripheral arterial disease, Ankle-brachial index, Interarm blood pressure difference, Obesity

## Abstract

**Background:**

The associations between obesity and abnormalities of upper and lower extremity arteries remain to be elucidated. This study is aimed to investigate whether general obesity and abdominal obesity are associated with upper and lower extremity artery diseases in a Chinese community population.

**Methods:**

This cross-sectional study included 13,144 participants in a Chinese community population. The associations between obesity parameters and abnormalities of upper and lower extremity arteries were evaluated. Multiple logistic regression analysis was used to assess the independence of associations between obesity indicators and abnormalities of peripheral arteries. Nonlinear relationship between body mass index (BMI) and risk of ankle-brachial index (ABI) ≤ 0.9 was evaluated using a restricted cubic spline model.

**Results:**

The prevalence of ABI ≤ 0.9 and interarm blood pressure difference (IABPD) ≥ 15 mmHg in the subjects was 1.9% and 1.4% respectively. Waist circumference (WC) was independently associated with ABI ≤ 0.9 (OR 1.014, 95% CI 1.002–1.026, *P* = 0.017). Nevertheless, BMI was not independently associated with ABI ≤ 0.9 using linear statistical models. Meanwhile, BMI and WC were independently associated with IABPD ≥ 15 mmHg respectively (OR 1.139, 95% CI 1.100–1.181, *P* < 0.001, and OR 1.058, 95% CI 1.044–1.072, *P* < 0.001). Furthermore, prevalence of ABI ≤ 0.9 was displayed with a U-shaped pattern according to different BMI (< 20, 20 to < 25, 25 to < 30, and ≥ 30). Compared with BMI 20 to < 25, risk of ABI ≤ 0.9 was significantly increased when BMI < 20 or ≥ 30 respectively (OR 2.595, 95% CI 1.745–3.858, *P* < 0.001, or OR 1.618, 95% CI 1.087–2.410, *P* = 0.018). Restricted cubic spline analysis indicated a significant U-shaped relationship between BMI and risk of ABI ≤ 0.9 (*P* for non-linearity < 0.001). However, prevalence of IABPD ≥ 15 mmHg was significantly increased with incremental BMI (*P* for trend < 0.001). Compared with BMI 20 to < 25, the risk of IABPD ≥ 15 mmHg was significantly increased when BMI ≥ 30 (OR 3.218, 95% CI 2.133–4.855, *P* < 0.001).

**Conclusions:**

Abdominal obesity is an independent risk factor for upper and lower extremity artery diseases. Meanwhile, general obesity is also independently associated with upper extremity artery disease. However, the association between general obesity and lower extremity artery disease is displayed with a U-shaped pattern.

## Introduction

Atherosclerotic cardiovascular diseases (ASCVDs) which may involve coronary artery disease (CAD), atherosclerotic cerebral infarction, peripheral arterial disease (PAD), and atherosclerotic changes in other arteries, are the main causes of mortality worldwide [1–4]. PAD may include arterial disease of lower extremities, upper extremities, renal artery, carotid artery, or other peripheral arteries, and is one of the manifestation of systemic atherosclerosis [5]. PAD is an important component of the ASCVD, but is often underestimated by cardiologists. In fact, PAD was associated with higher risk of all-cause and cardiovascular disease (CVD) mortality in Chinese patients with high cardiovascular risk in our previous studies [6, 7]. Ankle-brachial index (ABI) ≤ 0.90 can be considered as a criterion for the diagnosis of lower extremity PAD [8]. At the same time, increased interarm systolic blood pressure difference (IABPD) often signifies the potential abnormalities of upper extremity arteries mainly including subclavian artery, brachiocephalic trunk, and axillary artery [9, 10]. Previous studies revealed that lower ABI and higher IABPD were associated with increased mortalities respectively in Chinese [6, 11].

Obesity is associated with a much higher prevalence of comorbidities such as diabetes, hypertension, and metabolic syndrome, which then increase the risk of ASCVD. In addition, obesity may also be an independent risk factor for the development of ASCVD [12]. With the improvement of living standards and change of lifestyle, the prevalence of obesity has been significantly elevated in China in recent years. Thus, more attention should be paid to obesity related metabolic and cardiovascular disorders in China. Obesity can be classified as general obesity and abdominal obesity. However, the associations between various kinds of obesity and abnormalities of upper and lower extremity arteries remain to be elucidated to date. It is worth noting that the data on the relationship between body mass index (BMI) and abnormalities of lower extremity arteries are controversial. A previous study found that the risk of lower extremity PAD was increased with incremental BMI [13]. But another study indicated that BMI did not increase the risk of developing lower extremity PAD [14]. Meanwhile, the association between obesity and abnormalities of upper extremity arteries was rarely investigated in previous studies. Thus, this study is aimed to investigate whether general obesity and abdominal obesity are associated with the prevalence of upper and lower extremity artery disease in a community population in China.

## Materials and methods

### Study subjects

**Fig. 1:**
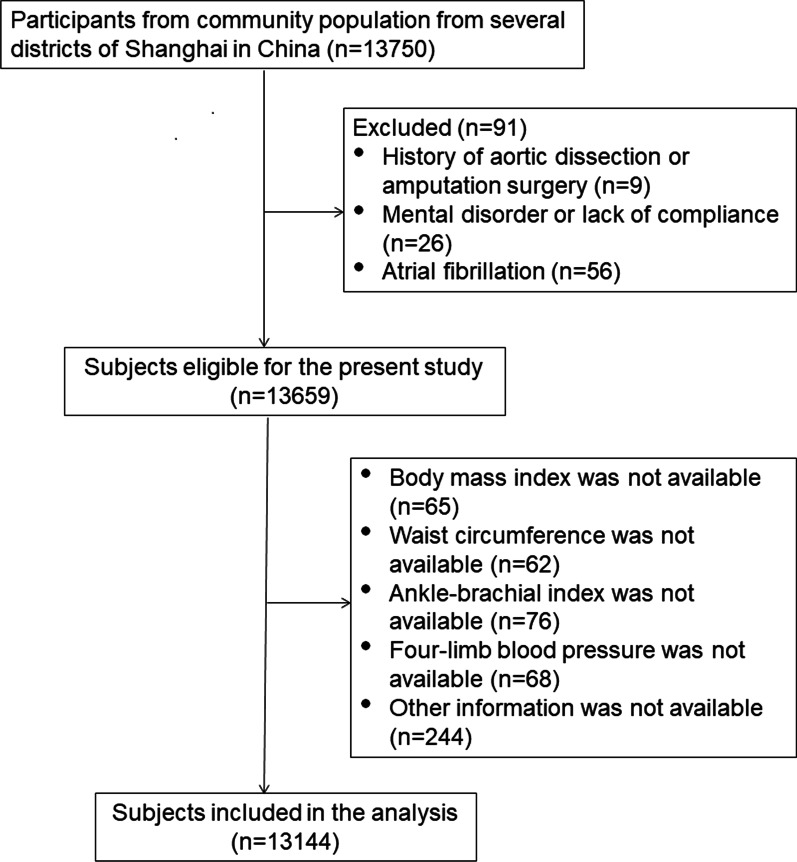
Flow chart of subjects enrollment

The study subjects (n = 13,750) were enrolled through cluster multistage and random sampling to community population from several districts of Shanghai in China in this cross-sectional study. The participants aged more than 18 years old were investigated in each center from May to September in 2016. Exclusion criteria included history of aortic dissection, history of amputation surgery, atrial fibrillation, mental disorder or lack of compliance. After the subjects with incomplete data or exclusion criteria were removed, there were totally 13,144 participants left (Fig. 1).

The study complied with the Declaration of Helsinki. It was also approved by the ethics committee of Shanghai Jiao Tong University and informed consent was obtained from all the participants prior to enrollment.

### Four-limb blood pressure and ABI measurement

Four-limb blood pressure and ABI measurement was performed by trained technicians using a non-invasive vascular profiling system (Omron VP-1000 vascular profiling system, Japan) [3]. This system ensured accurate and reliable ABI measurement using advanced oscillometric technology. Simultaneous blood pressure measurement at all four limbs was included, using a dual chamber cuff system and a proprietary algorithm. Measurement was performed after a 10-min rest in the supine position with the upper body as flat as possible. The device simultaneously and automatically measured the blood pressures twice, and then we calculated the means to get final blood pressure values. Bilateral ankle and brachial artery pressures, and bilateral ABI were supplied after measurement. ACC/AHA guidelines recommend ABI ≤ 0.90 as the criterion for the diagnosis of lower extremity PAD [8]. Meanwhile, IABPD ≥ 15 mmHg was considered as the potential abnormalities of upper extremity arteries according to literatures in this study [9, 10].

### Clinical data collection

A case report form was developed to record general characteristics, clinical diagnosis, and biochemical examination. Waist circumference (WC) was measured at the middle point between the costal margin and iliac crest. BMI was calculated as body weight in kilograms divided by body height in meters squared (kg/m^2^). Smoking habit was categorized as current smoking, ever smoking, or no smoking. Current smoking was determined when subjects were smoking currently and more than one cigarette daily in at least one year continuously. Ever smoking was determined when subjects smoked more than one cigarette daily, but had quitted smoking at least one year before. Drinking habit was categorized as current drinking, ever drinking, or no drinking. Current drinking was determined when subjects were drinking liquor, beer or wine currently in at least one year. Ever drinking was determined when subjects drank previously, but had quitted drinking at least one year before. History of lipid disorders included that plasma total cholesterol was ≥ 5.7 mmol/l, or low-density lipoprotein cholesterol (LDL-C) was ≥ 3.6 mmol/l, or high-density lipoprotein cholesterol (HDL-C) < 1.04 mmol/l, triglyceride was ≥ 1.7 mmol/l, or treatment with antihyperlipidemic agents due to hyperlipidemia. Hypertension was diagnosed by systolic blood pressure (SBP) ≥ 140 mmHg, or diastolic blood pressure (DBP) ≥ 90 mmHg, or being actively treated with anti-hypertension drugs. Diabetes mellitus was diagnosed by a fasting plasma glucose ≥ 7.0 mmol/l, or by a random plasma glucose ≥ 11.1 mmol/l, or when they were actively receiving therapy using insulin or oral medications for diabetes. Chronic kidney disease was defined as an estimated glomerular filtration rate (eGFR) < 60 ml/min/1.73 m^2^.

### Statistical analysis

Data entry and management were performed using Epidata software, version 3.1 (Epidata Association, Odense, Denmark). All statistical analyses were conducted with SPSS 22.0 (IBM, Armonk, NY, USA) and R language software (version 4.1.1). Continuous variables were expressed as the mean ± standard deviation, and categorical variables as frequencies (percentages). The chi-square test was used to compare categorical variables. The linear tendency was evaluated among several groups using trend test. The independent-sample t-test and one-way analysis of variance (ANOVA) were used to compare continuous variables among two or more groups. Multiple logistic regression analysis was used to assess the independence of the associations between obesity indicators and various abnormalities of peripheral arteries, and the odds ratio (OR) and 95% confidence interval (95% CI) was calculated. We also explored the nonlinear relationship between BMI and the risk of ABI ≤ 0.9 using a restricted cubic spline model by multivariable adjustment with three knots (at the 10th, 50th, and 90th percentiles). *P* < 0.05, which is two-sided, was considered significant.

## Results

### Study participants characteristics

General characteristics of the 13,144 participants by gender were shown in Table 1. The mean age was 52.2 ± 13.1 years old. 7181 subjects of them (54.6%) were man. The average BMI of all participants was 25.2 ± 3.81 kg/m^2^, and the average WC was 88.5 ± 11.7 cm respectively. The average ABI was 1.08 ± 0.09, and the average IABPD was 3.55 ± 3.79 mmHg respectively. Furthermore, the prevalence of ABI ≤ 0.9 and IABPD ≥ 15 mmHg in this study population was 1.9% and 1.4% respectively.

**Table 1:** Clinical characteristics of study participants according to gender

Variables	All (n = 13,144)	Man (n = 7181)	Woman (n = 5963)	*P* value
Age (years)	52.2 ± 13.1	51.7 ± 13.3	52.8 ± 13.0	< 0.001
BMI (kg/m^2^)	25.2 ± 3.81	25.5 ± 3.57	24.8 ± 4.05	< 0.001
WC (cm)	88.5 ± 11.7	91.0 ± 10.6	85.5 ± 12.2	< 0.001
Smoking	–	–	–	< 0.001
Current smoking (n, %)	3667 (27.9%)	3529 (49.1%)	138 (2.3%)	–
Ever smoking (n, %)	654 (40.1%)	619 (8.6%)	35 (0.6%)	–
No smoking (n, %)	8823 (40.1%)	3033 (42.2%)	5790 (97.1%)	–
Drinking	–	–	–	< 0.001
Current drinking (n, %)	2612 (19.9%)	2496 (34.8%)	116 (1.9%)	–
Ever drinking (n, %)	485 (3.7%)	463 (6.4%)	22 (0.4%)	–
No drinking (n, %)	10,047 (76.4%)	4222 (58.8%)	5825 (97.7%)	–
Diabetes mellitus (n, %)	1399 (10.6%)	805 (11.2%)	594 (10.0%)	0.021
Hypertension (n, %)	5720 (43.5%)	3040 (42.3%)	2680 (44.9%)	0.003
Lipid disorders (n, %)	6212 (47.3%)	4133 (57.6%)	2079 (34.9%)	< 0.001
Chronic kidney disease (n, %)	335 (2.5%)	143 (2.0%)	192 (3.2%)	< 0.001
Total cholesterol (mmol/l)	4.83 ± 1.04	4.82 ± 1.04	4.85 ± 1.04	0.289
Total triglyceride (mmol/l)	2.01 ± 1.82	2.22 ± 2.04	1.63 ± 1.25	< 0.001
LDL-C (mmol/l)	2.74 ± 0.90	2.74 ± 0.92	2.74 ± 0.86	0.737
HDL-C (mmol/l)	1.20 ± 0.32	1.13 ± 0.30	1.33 ± 0.31	< 0.001
Fasting plasma glucose (mmol/l)	5.36 ± 1.71	5.39 ± 1.74	5.31 ± 1.65	0.046
Serum creatinine (umol/l)	81.0 ± 38.0	86.8 ± 37.8	72.7 ± 36.6	< 0.001
eGFR (ml/min/1.73m^2^)	95.1 ± 22.3	97.0 ± 21.5	92.3 ± 23.2	< 0.001
ABI	1.08 ± 0.09	1.10 ± 0.09	1.07 ± 0.08	< 0.001
Systolic BP in left arm (mmHg)	130 ± 20.1	130 ± 18.0	130 ± 22.3	0.438
Diastolic BP in left arm (mmHg)	78.8 ± 12.2	80.3 ± 11.6	77.1 ± 12.6	< 0.001
Systolic BP in right arm (mmHg)	131 ± 20.0	131 ± 18.1	131 ± 22.1	0.706
Diastolic BP in right arm (mmHg)	79.4 ± 12.2	80.8 ± 11.7	77.6 ± 12.6	< 0.001
Systolic BP in left ankle (mmHg)	146 ± 26.3	147 ± 24.6	144 ± 28.0	< 0.001
Diastolic BP in left ankle (mmHg)	76.6 ± 12.2	77.9 ± 11.9	74.9 ± 12.4	< 0.001
Systolic BP in right ankle (mmHg)	148 ± 26.7	149 ± 25.1	146 ± 28.3	< 0.001
Diastolic BP in right ankle (mmHg)	76.3 ± 12.2	77.8 ± 12.0	74.6 ± 12.3	< 0.001
IABPD (mmHg)	3.55 ± 3.79	3.50 ± 3.97	3.61 ± 3.55	0.092
ABI ≤ 0.9	256 (1.9%)	120 (1.7%)	136 (2.3%)	0.012
IABPD ≥ 15 mmHg	180 (1.4%)	82 (1.1%)	98 (1.6%)	0.014

*BMI* body mass index, *WC* waist circumference, *BP* blood pressure, *LDL-C* low-density lipoprotein cholesterol, *HDL-C* high-density lipoprotein cholesterol, *eGFR* estimated glomerular filtration rate, *ABI* ankle-brachial index, *IABPD* interarm systolic blood pressure difference

Values are means ± SD, or numbers with percentage in parenthesis

### BMI and WC values according to different ABI and IABPD categories

The BMI and WC according to different ABI and IABPD categories were calculated and compared. WC was significantly higher in subjects with ABI ≤ 0.9 than that in subjects with ABI > 0.9 (*P* < 0.001, Table 2). However, BMI was not significantly different in subjects with ABI ≤ 0.9 and with ABI > 0.9 (*P* = 0.844, Table 2). At the same time, the WC and BMI were significantly higher in subjects with IABPD ≥ 15 mmHg than those in subjects with IABPD < 15 mmHg respectively (both *P* < 0.001, Table 2).

**Table 2:** BMI and WC values according to different ABI and IABPD categories

Variables	ABI > 0.9 (n = 12,888)	ABI ≤ 0.9 (n = 256)	*P* value	IABPD < 15 mmHg (n = 12,964)	IABPD ≥ 15 mmHg (n = 180)	*P* value
BMI (kg/m^2^)	25.2 ± 3.79	25.3 ± 4.75	0.844	25.2 ± 3.79	27.8 ± 4.66	< 0.001
WC (cm)	88.4 ± 11.6	91.0 ± 13.3	0.001	88.4 ± 11.6	97.9 ± 12.2	< 0.001

*BMI* body mass index, *WC* waist circumference, *ABI* ankle-brachial index, *IABPD* interarm systolic blood pressure difference

Values are means ± SD

### Independence of BMI and WC associated with different ABI and IABPD categories

In order to evaluate the independence of BMI and WC associated with different ABI and IABPD categories, multiple logistic regression analysis was used to calculate the OR and 95% CI of BMI and WC associated with ABI ≤ 0.9 and IABPD ≥ 15 mmHg respectively with adjustment for other potential confounders including age, men, smoking, drinking, hypertension, diabetes mellitus, lipid disorders, and chronic kidney disease. These indicators of obesity entered regression equation as continuous variables respectively. We found that WC was independently associated with ABI ≤ 0.9 (OR 1.014, 95% CI 1.002–1.026, *P* = 0.017, Table 3). Nevertheless, BMI was not independently associated with ABI ≤ 0.9 using this multiple logistic regression analysis. At the same time, the data showed that BMI and WC were independently associated with IABPD ≥ 15 mmHg respectively (OR 1.139, 95% CI 1.100–1.181, *P* < 0.001, and OR 1.058, 95% CI 1.044–1.072, *P* < 0.001, Table 3).

**Table 3:** Independence of BMI and WC associated with ABI ≤ 0.9 and IABPD ≥ 15 mmHg

Variables	OR	95% CI	*P* value
ABI ≤ 0.9	–	–	–
BMI (kg/m^2^)	0.999	0.965–1.034	0.954
WC (cm)	1.014	1.002–1.026	0.017
IABPD ≥ 15 mmHg	–	–	–
BMI (kg/m^2^)	1.139	1.100–1.181	< 0.001
WC (cm)	1.058	1.044–1.072	< 0.001

Multiple logistic regression analysis was used to calculate the odds ratio (OR) and 95% CI of body mass index (BMI), and waist circumference (WC) (independent variables) associated with ankle-brachial index (ABI) ≤ 0.9, or interarm systolic blood pressure difference (IABPD) ≥ 15 mmHg respectively with adjustment for other potential confounders including age, men, smoking, drinking, hypertension, diabetes mellitus, lipid disorders, and chronic kidney disease. BMI and WC entered regression equation as continuous variables respectively

### Prevalence of ABI ≤ 0.9 and IABPD ≥ 15 mmHg with different categories of BMI

**Fig. 2:**
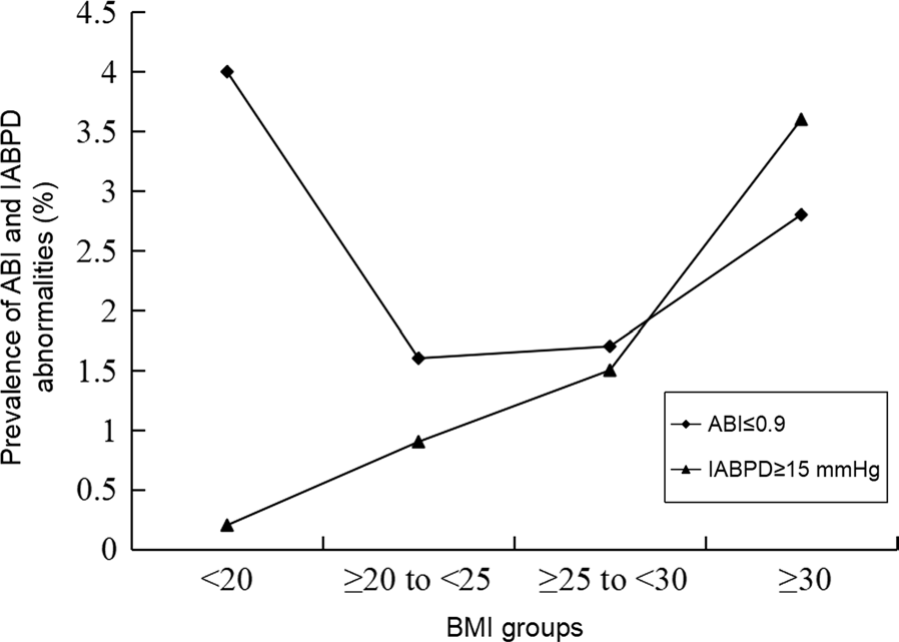
Prevalence of ABI ≤ 0.9 and IABPD ≥ 15 mmHg in different categories of BMI. ABI: ankle-brachial index; BMI: body mass index; IABPD: interarm systolic blood pressure difference. N = 925 for BMI < 20, 5643 for BMI ≥ 20 to < 25, 5203 for BMI ≥ 25 to < 30, and 1373 BMI ≥ 30. Prevalence of ABI ≤ 0.9 in subjects with BMI < 20 and BMI ≥ 30 was significantly higher compared with that in subjects with BMI ≥ 20 to < 25 respectively (both *P* < 0.001). Prevalence of IABPD ≥ 15 mmHg was significantly increased with incremental BMI (*P* for trend < 0.001)

As we mentioned in the above section, though we cannot discover a linear relationship between BMI and ABI statistically, we still try to explore the prevalence of ABI ≤ 0.9 in study subjects when they were categorized as four groups according to BMI (< 20, 20 to < 25, 25 to < 30, and ≥ 30). As a result, we found that prevalence of ABI ≤ 0.9 was displayed with a U-shaped pattern according to different BMI categories (Fig. 2). Prevalence of ABI ≤ 0.9 in subjects with BMI < 20 and BMI ≥ 30 was significantly higher compared with that in subjects with BMI 20 to < 25 respectively (both *P* < 0.001).

At the same time, we also tried to observe the prevalence of IABPD ≥ 15 mmHg when study subjects were categorized as four groups according to BMI. A different trend was discovered that prevalence of IABPD ≥ 15 mmHg was significantly increased with incremental BMI (*P* for trend < 0.001, Fig. 2).

### Relationship between BMI and abnormalities of peripheral arteries

The above data showed that, unlike WC, relationship between BMI and abnormalities of peripheral arteries appeared to be different. Thus, we further carefully evaluated whether various BMI categories (< 20, 20 to < 25, 25 to < 30, and ≥ 30) were associated with ABI ≤ 0.9 and IABPD ≥ 15 mmHg using multiple logistic regression analysis. The data displayed that, compared with BMI 20 to < 25, the risk of ABI ≤ 0.9 was significantly increased when BMI < 20 or ≥ 30 respectively (OR 2.595, 95% CI 1.745–3.858, *P* < 0.001, and OR 1.618, 95% CI 1.087–2.410, *P* = 0.018, Table 4). However, the risk of IABPD ≥ 15 mmHg tended to be increased when participants had bigger BMI. Compared with BMI 20 to < 25, the risk of IABPD ≥ 15 mmHg was significantly increased when BMI ≥ 30 (OR 3.218, 95% CI 2.133–4.855, *P* < 0.001, Table 4).

**Table 4:** Various categories of BMI associated with ABI ≤ 0.9 and IABPD ≥ 15 mmHg

Variables	OR	95% CI	*P* value
ABI ≤ 0.9	–	–	< 0.001
< 20	2.595	1.745–3.858	< 0.001
20 to < 25	Reference	Reference	Reference
25 to < 30	0.995	0.736–1.345	0.973
≥ 30	1.618	1.087–2.410	0.018
IABPD ≥ 15 mmHg	–	–	< 0.001
< 20	0.268	0.065–1.106	0.069
20 to < 25	Reference	Reference	Reference
25 to < 30	1.357	0.944–1.950	0.099
≥ 30	3.218	2.133–4.855	< 0.001

Multiple logistic regression analysis was used to calculate the odds ratio (OR) and 95% CI of body mass index (BMI) categories (< 20, n = 925; 20 to < 25, n = 5643; 25 to < 30, n = 5203; and ≥ 30, n = 1373), associated with ankle-brachial index (ABI) ≤ 0.9, or interarm systolic blood pressure difference (IABPD) ≥ 15 mmHg respectively with adjustment for other potential confounders including age, men, smoking, drinking, hypertension, diabetes mellitus, lipid disorders, and chronic kidney disease

**Fig. 3:**
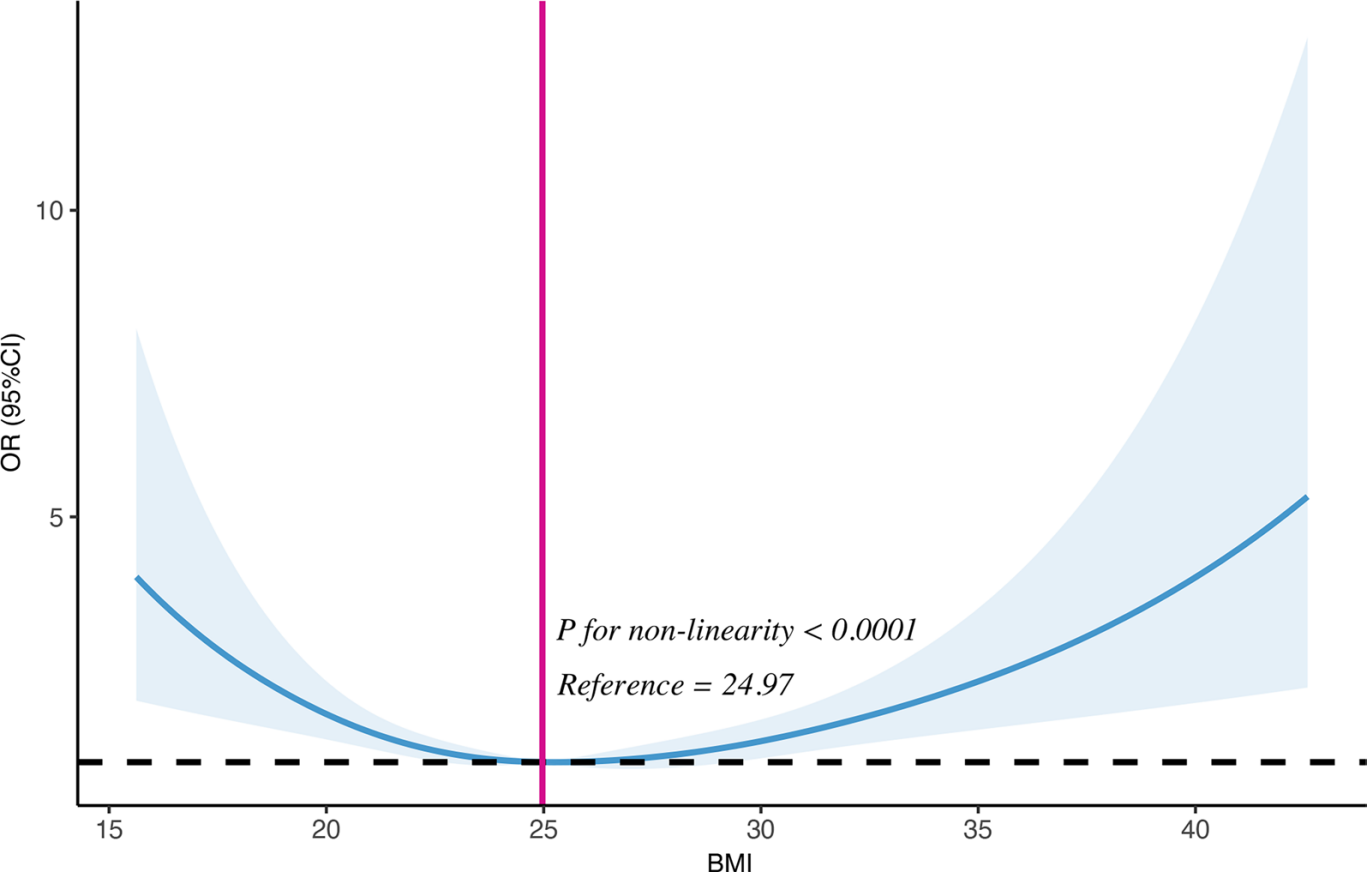
Nonlinear relationship between BMI and the risk of ABI ≤ 0.9. Y-axis stands for the odds ratio (OR) and 95% CI of body mass index (BMI) (independent variable) associated with ankle-brachial index (ABI) ≤ 0.9 using restricted cubic spline analysis, with adjustment for other potential confounders including age, men, smoking, drinking, hypertension, diabetes mellitus, lipid disorders, and chronic kidney disease. A significant U-shaped relationship between BMI and the risk of ABI ≤ 0.9 was exhibited (*P* for non-linearity < 0.001)

Furthermore, we also explored the nonlinear relationship between BMI and the risk of ABI ≤ 0.9 using a restricted cubic spline model by multivariable adjustment. Restricted cubic spline analysis (Fig. 3) indicated a significant U-shaped relationship between BMI and the risk of ABI ≤ 0.9 (*P* for non-linearity < 0.001).

## Discussion

The association between obesity and abnormalities of upper extremity arteries was rarely investigated in previous studies. The data in our study showed that both general and abdominal obesity parameters were independently associated with IABPD ≥ 15 mmHg respectively. A previous data demonstrated that BMI was in connection with IABPD ≥ 10 mmHg. However, WC was only statistically associated with inter-arm differences in diastolic blood pressure ≥ 10 mmHg [15]. The cut-off value of inter-arm differences was different from that in our study, and the sample size of the previous study was relatively small.

However, only abdominal obesity parameter was significantly higher in subjects with ABI ≤ 0.9. Meanwhile, BMI was not independently associated with ABI ≤ 0.9 using linear statistical models. In fact, previous studies also manifested that more patients with ABI ≤ 0.9 had abdominal obesity than those without lower extremity PAD [16]. Lots of data demonstrated that abdominal obesity was an independent risk factor for the development of ASCVD including PAD [12, 16]. These were possibly because the people with abdominal obesity tended to have more atherosclerotic plaques in arteries. Nevertheless, previous study provided controversial data on association between BMI and ASCVD including lower extremity PAD [17]. More abundant data on both abdominal obesity and general obesity associated with lower extremity PAD were supplied in our study, and we think the data in this study can be helpful to explore the relationship between obesity and lower extremity PAD.

In order to explore the relationship between BMI and lower extremity PAD further, subjects in this study were categorized as four groups according to BMI. As a result, we found that compared with BMI 20 to < 25, the risk of ABI ≤ 0.9 was significantly increased by more than 2.5-fold and 1.6-fold when BMI < 20 or ≥ 30 respectively. Additionally, a significant U-shaped relationship was observed between BMI and the risk of ABI ≤ 0.9 using restricted cubic spline analysis, which indicated that the risk of ABI ≤ 0.9 increased when BMI exceeded or less than the median value (i.e., 24.97). These data manifested that it was not a linear relationship, but a U-shaped pattern between BMI and ABI ≤ 0.9 in this Chinese population of our study. We speculated that when subjects had bigger BMI, they would possibly have more atherosclerotic plaques in lower extremity arteries. However, this study showed that underweight subjects also had increased prevalence of ABI ≤ 0.9. Similar results were found in a previous study [18], but the reason is not very clear yet. Some researchers considered that the underweight patients possibly had higher levels of inflammation which might promote the development of atherosclerosis [19]. In fact, a phenomenon called obesity paradox showed that a low body weight was also associated with cardiovascular disease and mortalities [20]. A previous study manifested that obesity was associated with lower in-hospital mortality in PAD patients relative to those with normal-weight/over-weight. This obesity survival paradox was independent of age, gender and comorbidities and observed for all obesity classes [21]. However, the precise mechanism is still not clear. We think that the obesity paradox between BMI and ABI needs to be further studied.

We not only studied the relationship between general obesity and lower extremity PAD, but also studied the relationship between general obesity and upper extremity PAD in this study. Univariate analysis and multiple logistic regression analysis indicated that prevalence of IABPD ≥ 15 mmHg was significantly increased with incremental BMI. This data was quite different according to the above analysis on the relationship between general obesity and ABI ≤ 0.9. However, the causes for this discrepancy were unknown. We speculated the possible causes as follows. First, ABI ≤ 0.90 can be considered as the presence of lower extremity PAD. However, IABPD ≥ 15 mmHg possibly signifies abnormalities in upper extremity arteries mainly including subclavian artery, brachiocephalic trunk, and axillary artery [10]. Risk factors for abnormalities of arteries at different anatomical locations might be different. Second, though atherosclerosis is the main cause of the upper or lower extremity PAD, there are also other divergent causes. Lower extremity PAD might be caused by atherosclerosis, takayasu arteritis, and so on. Meanwhile, more causes of the upper extremity PAD were found such as atherosclerosis, thoracic outlet syndrome, giant cell arteritis, takayasu arteritis, radiation artery fibrosis, fibromuscular dysplasia, and so on [10]. In fact, atherosclerosis in lower extremity PAD is possibly more frequently to be found compared with that in upper extremity PAD [10, 11]. The associations of these divergent pathogenic risk factors with BMI appear to be more complex. Of course, the future studies are still needed to explain the discrepancies.

In summary, this study demonstrates that abdominal obesity is an independent risk factor for abnormalities of upper and lower extremity arteries. Meanwhile, general obesity is also independently associated with abnormalities of upper extremity arteries. However, the association between general obesity and lower extremity PAD is displayed with a U-shaped pattern. We believe that the exploration of risk factors for abnormalities of upper and lower extremity arteries, and then the comprehensive control of abdominal obesity, will possibly help to improve the understanding and control of PAD.

## Data Availability

The datasets in the current study are available from the corresponding author on reasonable request.

## Abbreviations

ASCVD: Atherosclerotic cardiovascular disease
CAD: Coronary artery disease
PAD: Peripheral arterial disease
CVD: Cardiovascular disease
ABI: Ankle-brachial index
IABPD: Interarm systolic blood pressure difference
WC: Waist circumference
BMI: Body mass index
LDL-C: Low-density lipoprotein cholesterol
HDL-C: High-density lipoprotein cholesterol
BP: Blood pressure
SBP: Systolic blood pressure
DBP: Diastolic blood pressure
eGFR: Estimated glomerular filtration rate
ANOVA: One-way analysis of variance
OR: Odds ratio
95% CI: 95% Confidence interval
